# Electronic Structure, Dielectric Response, and Surface Charge Distribution of RGD (1FUV) Peptide

**DOI:** 10.1038/srep05605

**Published:** 2014-07-08

**Authors:** Puja Adhikari, Amy M. Wen, Roger H. French, V. Adrian Parsegian, Nicole F. Steinmetz, Rudolf Podgornik, Wai-Yim Ching

**Affiliations:** 1 Department of Physics and Astronomy, University of Missouri-Kansas City, Kansas City, MO, 64110, USA; 2 Department of Biomedical Engineering, Case Western Reserve University, 10900 Euclid Avenue, Cleveland, OH 44106, USA; 3 Department of Materials Science and Engineering, Case Western Reserve University, 10900 Euclid Avenue, Cleveland, OH 44106, USA; 4 Department of Physics, Case Western Reserve University, 10900 Euclid Avenue, Cleveland, OH 44106, USA; 5 Department of Physics, University of Massachusetts, Amherst, Massachusetts 01003, USA; 6 Department of Radiology, Case Western Reserve University, 10900 Euclid Avenue, Cleveland, OH 44106, USA; 7 Department of Macromolecular Science and Engineering, Case Western Reserve University, 10900 Euclid Avenue, Cleveland, OH 44106, USA; 8 Department of Theoretical Physics, J. Stefan Institute, SI-1000 Ljubljana, Slovenia; 9 Department of Physics, Faculty of Mathematics and Physics, University of Ljubljana, SI-1000 Ljubljana, Slovenia

## Abstract

Long and short range molecular interactions govern molecular recognition and self-assembly of biological macromolecules. Microscopic parameters in the theories of these molecular interactions are either phenomenological or need to be calculated within a microscopic theory. We report a *unified methodology* for the *ab initio* quantum mechanical (QM) calculation that yields all the microscopic parameters, namely the partial charges as well as the frequency-dependent dielectric response function, that can then be taken as input for macroscopic theories of electrostatic, polar, and van der Waals-London dispersion intermolecular forces. We apply this methodology to obtain the electronic structure of the cyclic tripeptide RGD-4C (1FUV). This *ab initio unified methodology* yields the relevant parameters entering the long range interactions of biological macromolecules, providing accurate data for the partial charge distribution and the frequency-dependent dielectric response function of this peptide. These microscopic parameters determine the range and strength of the intricate intermolecular interactions between potential docking sites of the RGD-4C ligand and its integrin receptor.

Nature has evolved many sophisticated bio-specific recognition systems that play a crucial role in cell signaling and orchestration of self-assembly of molecules, cells, and entire organisms. Fundamental understanding of the molecular machinery governing these bio-specific interactions is expected to have impact on the basic sciences, materials science, and translational approaches where bio-recognition systems are being exploited and further developed to yield novel functional materials with various properties such as self-assembly, stimuli-response, and/or self-healing.

Interactions between biological macromolecules can be deduced from standard principles of colloid and nanoscale stability theory^1^ that identify different types of direct *long range* interactions as well as different types of *short-range* solvent-mediated interactions, together governing the molecular recognition and self-assembly of biological macromolecules^2^. The former include *electrostatic interactions*^3^, depending on the specific nature of molecular charges and the net charge on a body, *polar interactions*^4^ arising from dipolar and higher order charge multipoles, and *van der Waals-London dispersion (vdW) interactions*^4^, that in their turn depend on the details of the dielectric response properties of the molecular materials. The short-range solvent-mediated components of the overall intermolecular forces can be classified as *hydration interactions*^5^, due to hydrophilic moieties, and *hydrophobic interactions*^6^, engendered by the hydrophobic moieties, both along the solvent-exposed surfaces.

Theoretical modeling of the long range components of the molecular interactions can be decomposed conceptually and methodologically into a *microscopic* and a *macroscopic* part^2^. While the two methodological levels are coupled in principle, the standard assumption is that this coupling is weak and that the microscopic calculation yields parameters for *isolated molecules* that then enter the macroscopic theory of interactions *between the molecules*. For electrostatic and vdW interactions, the microscopic part follows from *ab initio* QM calculations, that are in general of different types, focused specifically on the electronic structure calculations, which ideally yield the partial charges of all the atoms composing the interacting molecules, or indeed on the frequency-dependent dielectric function (the optical properties) of the whole molecule, respectively^7^. These microscopic parameters then enter the macroscopic theories, *viz.* the Poisson-Boltzmann (PB) theory and/or its derivatives for electrostatic^8^ and polar interactions^9^, and the Lifshitz theory for vdW interactions^4^, that provide the separation and angular dependence of the long range part of the interaction between the molecules^10^,^11^,^12^, governing important aspects of the recognition and assembly processes of macromolecules.

As a model for a biomolecular recognition system we chose a ligand-receptor couple, specifically, the well-known and extensively studied RGD peptide ligand-integrin receptor pair. However, one should note here, that these studies are available only in the setting of biochemistry, nanotechnology, and medicine, while no quantitative assessment of its electronic structure, optical properties, and partial charge has been undertaken. The tripeptide RGD plays a pivotal role in cell signaling. It should be noted that out of 24 known integrins, a third recognize and bind to the RGD tripeptide^13^. Receptor specificity and affinity is dependent on peptide conformation and flanking amino acids^14^. RGD-containing biomolecular materials have become popular as scaffolds for bone, tissue, and cartilage synthesis^15^; they are also candidates for radiotracers in imaging^16^,^17^,^18^ and targeted drug delivery^19^,^20^,^21^. The broader opportunities represented by molecular recognition systems, such as the ligand-receptor pairs of the RGD-integrin type, include also the ability to architecturally predefine building blocks that can then establish bonds between complementary surface moieties. Other bio-specific recognition systems, for example, are orthogonal pairs of coiled-coil peptides, which are being investigated for self-induced assembly of nanoparticles and hierarchical structures^22^,^23^. This type of remote control of nanoscale and mesoscale assembly and architecture is one of the opportunities recently identified in a mesoscale science report^24^, where programmable molecular recognition could be easily exploited for electrical energy storage, lighting, photovoltaic devices, and electronics.

In this paper, we report an *ab initio unified methodology* for the QM calculation of one representative isomer of RGD-4C (Figure 1) that yields its electronic structure, its partial charges, as well as its frequency-dependent dielectric response function, in a single scalable calculation that could then be taken as input for the macroscopic PB-vdW theories of intermolecular forces. RGD-4C is structurally characterized as a peptide with the amino acid sequence ACDCRGDCFCG consisting of alanine (A), cysteine (C), asparagine (D), glycine (G), phenylanine (F), and arginine (R). This peptide, identified through the phage library display^25^, is probably one of the most utilized RGD variants. There are four cysteines in RGD-4C, which would allow for three possible fully disulfide-bonded forms. Others have reported that only two forms, specifically the 1–4; 2–3 and 1–3; 2–4 bonded arrangements, are detected in the spontaneously cyclized peptide^26^. The 1–4; 2–3 bonded peptide, isomer RGD-A, is a stronger α_v_β_3_ integrin binder. It thus seemed appropriate to consider RGD-A as a paradigm for our calculations. To the best of our knowledge, there have been no fundamental studies on RGD peptides of this particular type.

While the *ab initio unified methodology* of our single scalable calculation yields accurate data for the partial charge distribution and the frequency-dependent imaginary part of the dielectric response function of this important peptide, this fundamental understanding in turn provides novel and helpful information for elucidating the nature of intricate intermolecular interactions between potential docking sites. In recent years, such calculations have started to emerge on various levels, with varied methodologies^27^, and are expected to make a major impact in biomedical engineering^28^,^29^,^30^,^31^,^32^, energy, and broadly in mesoscale science in general^24^.

## Results

**Figure 1:**
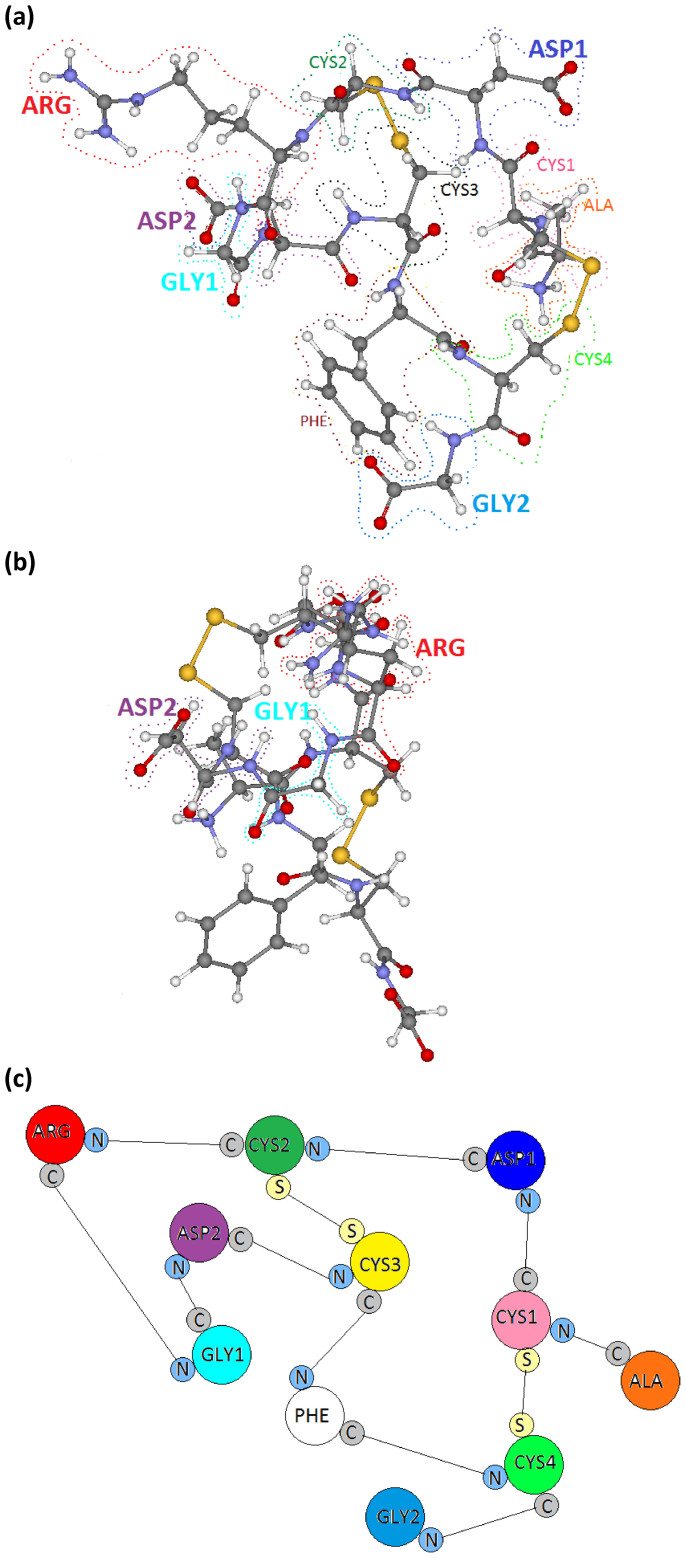
(a) Molecular structure of the peptide RGD-A. Each amino acid is enclosed by dashed line and marked with ARG, GLY and ASP in larger font since they represent the R,G,D respectively in the peptide; (b) same as (a) with 90 degree orientation; (c) Gross sketch of the structure of RGD-A showing two-looped structure and the connecting atoms in each amino acid.

The structure for RGD-A (1FUV) was obtained from the RCSB protein data bank (PDB) (http://www.rcsb.org/pdb/explore.do?structureId=1FUV, date accessed 5/23/2014) based on data from nuclear magnetic resonance (NMR) measurements. There are a total of 19 such models with the same number of atoms, molecular formula, molecular composition, and molecular weight but with a slightly different molecular volume. We selected model 1 for the electronic structure calculations. It has 11 amino acids that consist of a single ALA, ARG and PHE, two ASP and GLY, and 4 CYS AAs (altogether 135 atoms). The atomic positions of the amino acids of the RGD-A peptide were kept in a super-cell of size 26Å × 25Å × 20Å, which is sufficiently large to avoid any interaction with the same molecule in the adjacent cells. This model structure was then further relaxed using Vienna *ab-initio* simulation package (VASP) (see Methods). There was no significant change in the structure from PDB data, signifying the integrity and accuracy of the structure deposited in PDB. Figures 1 (a) and (b) show the molecular structure of the peptide with the constituting 11 amino acids delimited by dotted lines. Figure 1 (c) is a simplified symbolic sketch of the molecule showing two topological loops on the graph of chemical bonding for this peptide.

**Figure 2:**
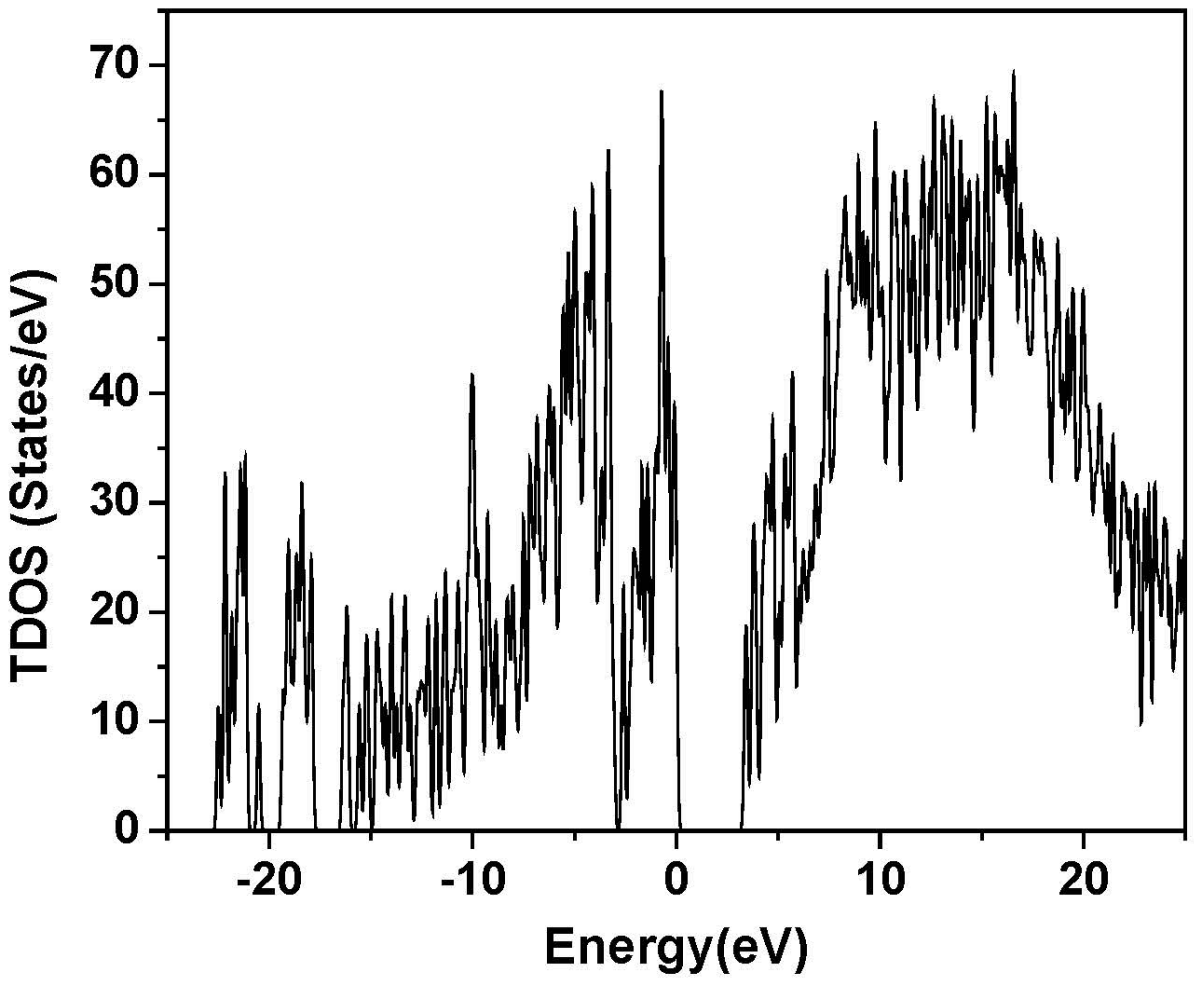
The calculated total density of states (TDOS) of 1FUV (RGD-A) with each energy state slightly broadened; the energy of HOMO state is set at 0.0 eV.

**Figure 3:**
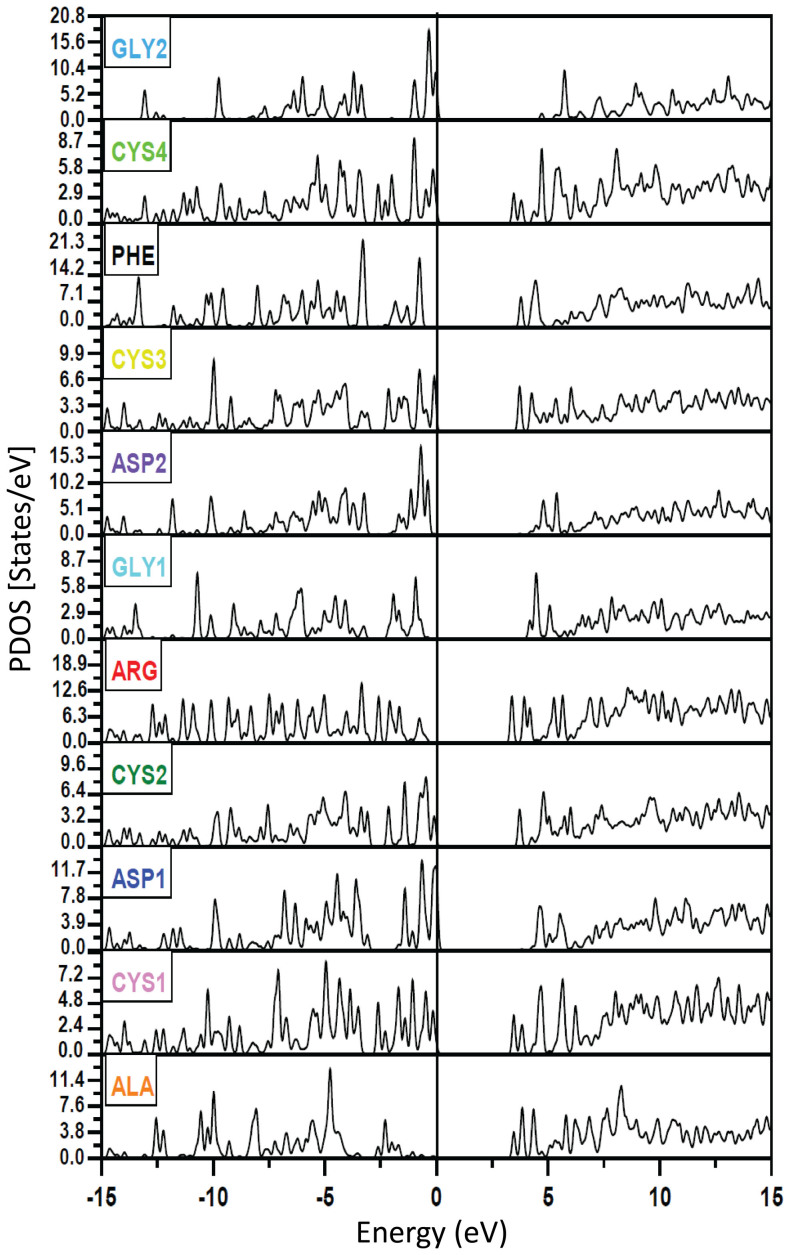
The calculated partial density of states (PDOS) for each amino acid as marked in each panel for 1FUV (RGD-A). Note the Y-axis scales for the amino acids are not the same for better visual juxtaposition.

Figure 2 shows the calculated total density of states (TDOS) or the number of electron states per unit of energy of RGD-A (1FUV). The HOMO-LUMO gap of 1FUV is about 3.38 eV and the range of the occupied valence band region is 22.9 eV. The TDOS is resolved into 11 partial DOS (PDOS) in Figure 3 for the six different types of amino acids in the sequence. The integrated area of the PDOS up to HOMO corresponds to the total number of electrons in the amino acid in the RGD molecule. The HOMO state (defined as the top of the valence band at 0.0 eV) originates from ASP1; the LUMO state is from ARG. An interesting feature in the PDOS is the four different CYS amino acids that have somewhat different features indicating the difference in their connectivity (1–4; 2–3) and in their local environments. The same can be said about the two different GLY (GLY1 and GLY2) and the two different ASP (ASP1 and ASP2) (see Figure 3). These differences for the same amino acid types are usually observed in the form of a shift of their peak positions in the PDOS spectra and their relative heights. Such differences reflect the ability of *ab initio* calculations in delineating the electron structures and local geometries of individual amino acids.

**Figure 4:**
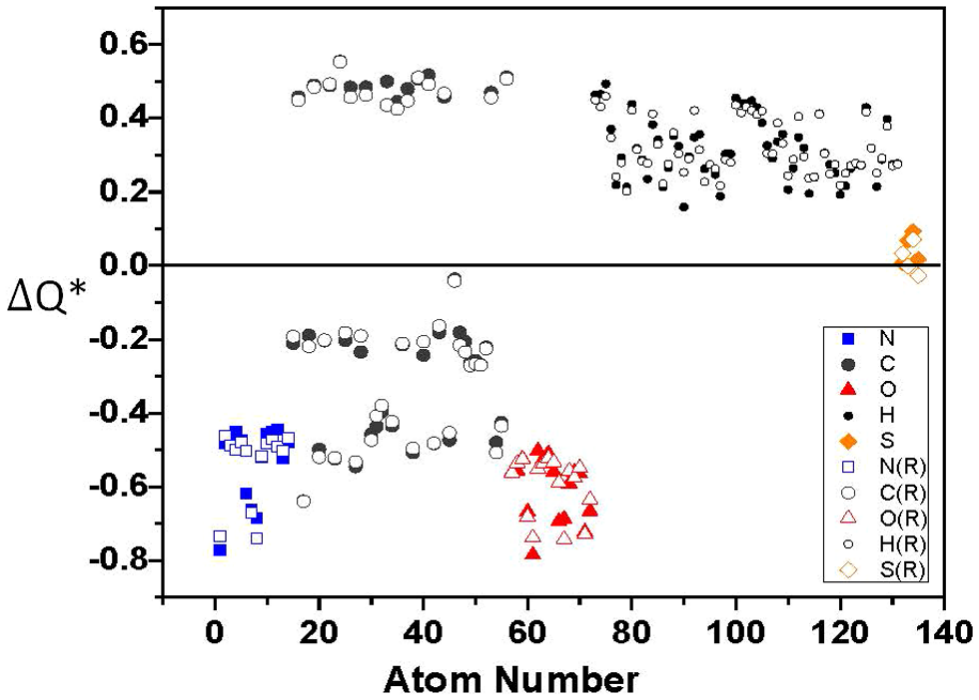
Calculated partial charge ΔQ* vs. atom number in 1FUV (RGD-A). Solid symbols are the values obtained from the calculation using the unrelaxed structure from PDB and the open symbols are from the VASP-relaxed structure (R).

Figure 4 displays the calculated partial charge ΔQ* on each of the 135 atoms in RGD-A (1FUV) for the relaxed structure (open symbol) and the original structure from PDB (closed symbol). Partial charge ΔQ* = Q^0^ − Q* is the deviation between the charge on the neutral atom Q^0^ and the effective charge of the same atom in the present calculation in accordance with the *Mulliken scheme*^33^ using the minimal basis in the *orthogonalized linear combination of atomic orbitals* (OLCAO) method^7^ (see Methods section). There is no significant change in the partial charge of the atoms before and after relaxation, indicating that the changes due to relaxation are minor. Nitrogen and oxygen atoms are all negatively charged, whereas all hydrogen atoms are positively charged and sulfur atoms are only slightly positive. However, the carbon atoms can be both positively and negatively charged according to their local coordination and bonding within different amino acids. The most positive ΔQ* value is 0.553 *e* which comes from a C atom in ASP1 and the most negative ΔQ* value for C is −0.640 *e* from a C atom in ALA. The most negative ΔQ* value is −0.741 *e* from the N atom of ARG and the O atom of ASP2 (−0.742 *e*). By adding the ΔQ* values of all the atoms within each amino acid, we obtain the partial charges for each amino acid.

**Figure 5:**
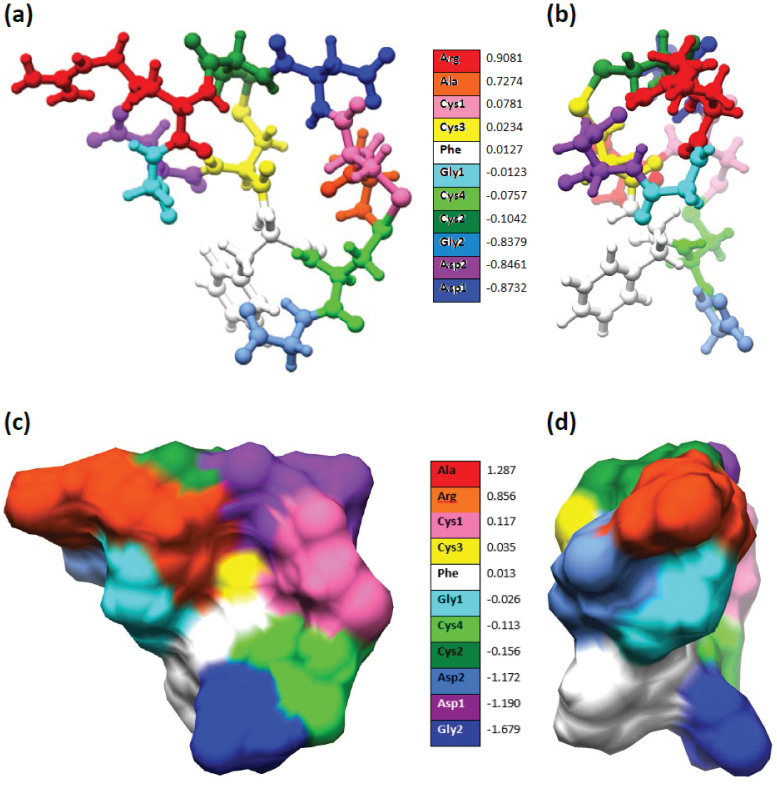
(a) Colored representation of the calculated partial charge in the ball-stick model of 1FUV (RGD-A). The actual values of the partial charge for the amino acids are listed in the color bar. (b) Rotation of figure (a) by 90°. (c) Surface charge density in solvent-excluded model of 1FUV (RGD-A). The actual values for the surface charge density in the unit of *e*/(nm)^2^ for the amino acids are listed in the color bar. (d) Rotation of figure (c) by 90°.

Figure 5 (a) and (b) presents a ball and stick model in two different orientations in which the amino acids are differentiated by color according to their partial charge. ASP has the most negative charge of −0.87 *e* (dark blue) whereas ARG has the most positive charge of +0.91 *e* (dark red). The variations in partial charge among amino acids (hence difference in color) exemplify the different chemical bonding between atoms within it. For instance, there are four cysteines, and their partial charges are −0.076 *e*, +0.023 *e*, −0.104 *e*, and +0.078 *e* for CYS4 (10), CYS3 (8), CYS2 (4), and CYS1 (2) respectively, where the integer in the parentheses indicates their location in the sequence.

At physiological pH 7.4, one standardly considers the following isolated amino acids as charged: aspartic acid (ASP) and glutamic acid (GLU) carrying a charge of −1.0 *e* originating from the deprotonated carboxylate on the side chains of aspartic and glutamic acid, lysine (LYS) and arginine (ARG) carrying a charge of +1.*0 e* originating in the protonated amine group of arginine and lysine, and histidine (HIS) carrying a fractional charge of +0.1 *e* originating in the protonated state of the secondary amine of histidine. Cysteine (CYS) is usually not considered to be an acid because the thiol group is often reactive and can form disulfide bonds^34^. The *ab initio* result that shows the most positive charge +0.91 *e* on ARG is in agreement with the above standard value of +1.0 *e*. Also in agreement, ASP with −0.87 *e* emerges as the most negatively-charged amino acid and compares well with −1.0 *e* assumed in the aqueous solvent at physiological conditions. The other amino acids usually assumed to be charged at physiological conditions are GLU with partial charge of −1.0 *e*, and LYS and HIS, the former with a partial charge of +1.0 *e and the latter with* +0.1 *e*. However, these amino acids are not part of the RGD peptide studied in this paper. On the other hand, the *ab initio* partial charges on ALA with +0.72 *e* and GLY with −0.84 *e* are quite substantial, while they are usually assumed to be neutral at physiological conditions. These differences between the *ab initio* and the standard values of partial charges imply that effects of the *solvent and local microenvironment*, such as proximity of other charged groups, geometry of the folded polypeptide, solution ions, dielectric permittivities of the various peptide moieties etc. all strongly affect the local environment of the dissociable amino acid groups in the polypeptide. They can have a dominating effect on its effective charge (for details see Ref. 35).

Apart from the assignment of partial charges, what is important in large macromolecules are their surface charge densities that determine the electrostatic potential and the long range polar interactions. Different amino acids have different effective volumes and surface areas based on the standard solvent excluded layer. Figure 5 (c) and (d) shows the distribution of the surface charge density in units of *e*/(nm)^2^ obtained by using the surface charge density for each amino acid normalized by its solvent excluded surface area. The surface charge density map shows the spectacular polar nature of the RGD-A (1FUV) peptide, which controls docking to its binding site (the integrin receptor) and its movements within the aqueous solution. The effective surface charge density is displayed in two orientations as in Figure 5 (a) and (b). Because of the local geometry of the folded peptide chain, the highest negative charge density is now exhibited by GLY2 (−1.679 e/(nm)^2^), with the highest positive charge density found on ALA (+1.287 e/(nm)^2^). The region close to PHE is more or less neutral (white color). The RGD motif exhibits the following effective surface charges: ARG with +0.856 e/(nm)^2^, GLY1 −0.026 e/(nm)^2^, and ASP2 −1.172 e/(nm)^2^.

The values of the surface charge density are not far from the values typically assumed for polypeptides (~0.6 *e*/(nm)^2^) and are smaller than that for DNA (~1 *e*/(nm)^2^) or many lipid bilayers (~0.1 to ~1 *e*/(nm)^2^) considered within idealized cylindrical or planar effective shape models. In fact, one should note that the charge density calculated here pertains to a certain choice of the area normalization, *i.e.*, solvent excluded in this case. In case one wants to use an effective geometric model of the molecule, e.g. spherical, cylindrical, prolate or oblate idealized forms, due provision must be taken into account for additional surface area normalization^36^. Nevertheless, the effective surface charge density shown in Figure 5 (c) and (d), rigorously calculated by QM means, does provide a quantitative measure for the charge multipoles associated with the molecule. The dipole is the most prominent, as it clearly shows the lacunae of the positive (and/or negative) charges that would provide molecular interaction handles to position the molecule on approach to another molecule.

**Figure 6:**
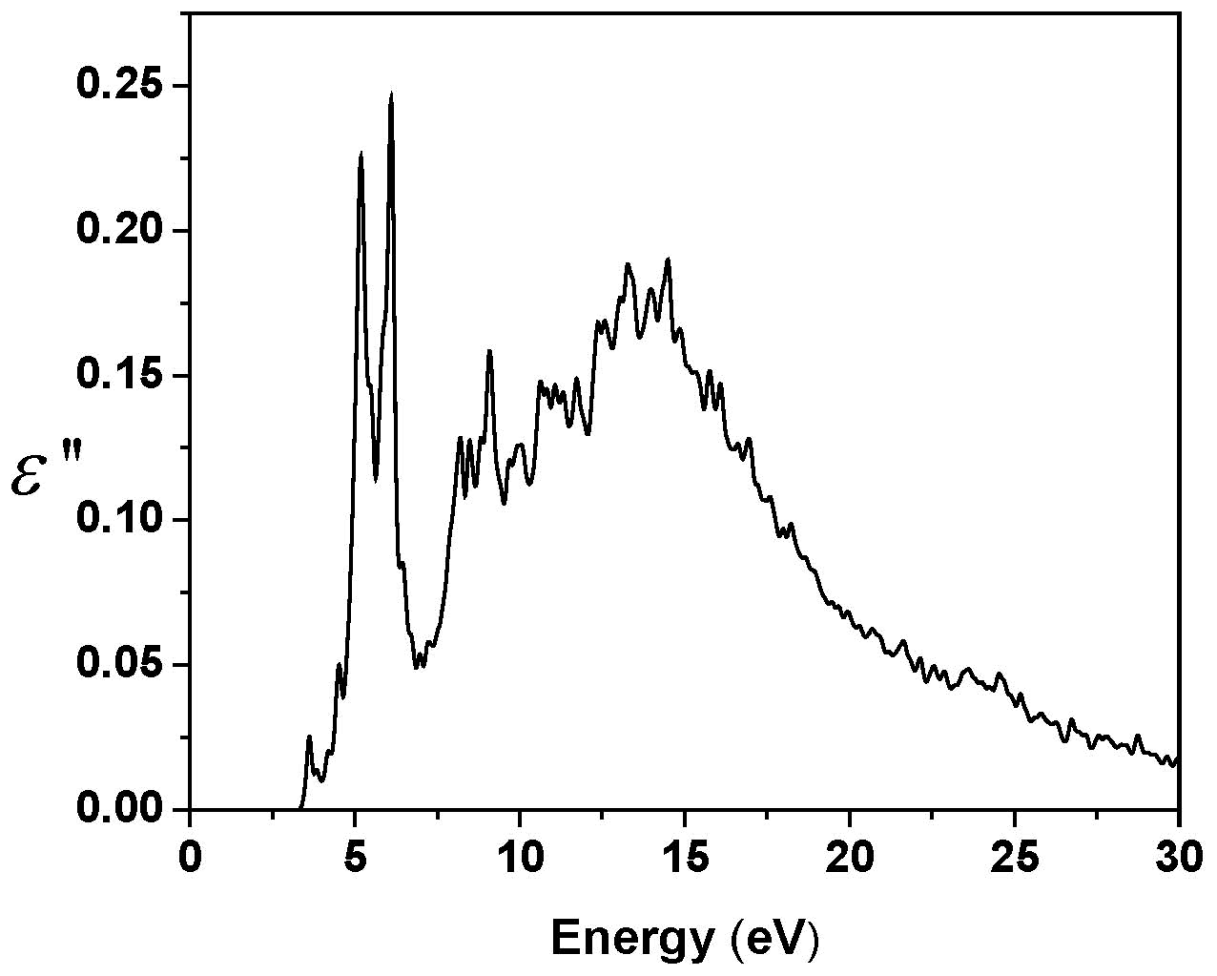
The imaginary part of the complex dielectric function, ε″, vs. optical transition energy of 1FUV (RGD-A).

In Figure 6, we show the calculated imaginary part of the dielectric response function as a function of frequency (in energy units), 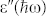, for RGD-A (1FUV) obtained according to Eq. (2) (see Methods section) using the *ab initio* wave functions for optical transitions from the occupied states to the unoccupied states fully including the effects of the dipole matrix elements. Apart from the generic broad peak at about 15 eV stemming from the bulk covalent interatomic bonding, it also prominently shows a spectacular sharp peak structure at 5.18 eV (Peak 1) and 6.10 eV (Peak 2), which correspond to wavelengths of 240 nm and 204 nm, respectively, that have not been seen in calculated optical spectra of any biomolecule yet. Such well defined sharp structures should be easily detectable. We can approximately trace the source of these transitions for different amino acids from the peak separations in occupied and unoccupied PDOS respectively, which could yield valuable insights into the functioning of this particular peptide. From Figure 3, it appears that Peak 1 can be associated with transitions in PHE and CYS1 whereas Peak 2 is composed of transitions in ASP2, GLY2, ALA, and CYS1. In general, different side groups of single amino acids contribute to the absorbance and are most prominent in the range of 250–280 nm, which may be indicated by the 5.18 eV peak. The absorbance for a complete peptide on the other hand, lies in the 190–230 nm range, and could therefore match the 6.10 eV peak.

## Discussion

We have studied the electronic structure, the partial surface charge distribution, and the imaginary part of the frequency-dependent dielectric response function in the RGD-4C peptide, specifically for the RGD-A isomer (1FUV), based on a rigorous *ab initio unified methodology* QM calculations. The results show that it has a HOMO-LUMO gap of about 3.38 eV. The real HOMO-LUMO gap could be a little larger since density functional theory (DFT) calculations using LDA approximation generally underestimate the band gap, as is well known and discussed in Ref. 7. In principle, one can get a slightly larger gap by using a different exchange-correlation potential in DFT, such as PAW-PBE or GGA, meta-GGA, or a hybrid potential such as B3LYP etc., depending on the system under investigation and on the basis set used in a particular method. LDA is currently implemented in the OLCAO method that we use. Since no calculation of the RGD electronic structure is available, it seems important to us to obtain a solid estimate on the HOMO-LUMO gap that can later be improved, if it turns out to be necessary. One thing is, nevertheless, certain: the HOMO-LUMO gap does not in any way affect the partial charge calculations which are derived from the occupied states well described by DFT. It may shift the two prominent peaks in the imaginary part of the dielectric response function, Figure 6, to a slightly higher frequency, though.

By resolving the total density of states of the peptide into components from individual amino acids, detailed interactions between them at an atomic level can be further elaborated. The surface charge density distribution obtained from partial charge calculations shows the molecule to be highly polar, thus able to promote interactions with its α_v_β_3_ integrin receptor either via long range polar complementarity or short-range hydrogen bonding.

We have also calculated the optical properties of this peptide. These show a striking feature of two sharp absorption peaks apart from the bulk broad interatomic bonding peak. The optical spectra of nano-scale objects, such as single wall carbon nanotubes (SWCNT) with different chirality, have already been used to estimate the long-range vdW interaction either in vacuum or in the solvent through their connection with Hamaker coefficients that quantify the strength of this interaction^10^,^11^,^12^. It is conceivable that the unique optical spectrum of RGD peptide could result in a strong vdW attractive force that could influence its interaction with other molecular moieties and even modify its mobility in aqueous solutions.

The *ab initio unified methodology* QM calculations used in this paper, which yield different microscopic parameters in a single scalable calculation, are the first for the RGD peptide to allow for a reliable estimate of the partial charges and the frequency dependent dielectric response function directly from the wave functions (see e.g. Eq. (1)) and can be further refined and applied to other substantially larger and significantly more complex biomolecules and proteins. They can also be used to refine the crucial parameters in molecular dynamic simulations vastly used in biophysics community. In view of the paucity of theoretical studies of partial charge distribution and dielectric response properties for the RGD peptide, we believe our seminal study will be helpful making simulation force field parameters more accurate and useful.

Future investigations should include in particular the investigation of solvent effects by adding explicit water molecules with and without salts and observing their influence on the electronic structure, surface charge distribution, and dielectric response, as well as using the present results to estimate the electrostatic and polar interactions between RGD and other biomolecules or peptides. One should nevertheless note, that such full scale quantum calculations are a non-trivial task, and even calculations with no explicit water molecules for this polypeptide have not been available before. In addition to the explicit solvent, one would also need to implement the microenvironment of the various dissociable groups on the solvent exposed surface of the polypeptide, making hopes for a full quantum mechanical treatment very remote.

## Methods

We used the orthogonalized linear combination of atomic orbitals (OLCAO) method^7^. The fundamental quantities discussed here are total density of states (TDOS), partial density of states (PDOS), and effective charge Q_α_* (for details see Ref. 7): 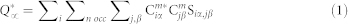
Here, the S_iα, jβ_ are the overlap integrals between the i^th^ orbital in the α^th^ atom and j^th^ orbital in the β^th^ atom and C^m^_jβ_ are the eigenvector coefficients of the m^th^ band, j^th^ orbital in the βth atom^7^. Basically, OLCAO is an electronic structure method based on DFT with local density approximation originally designed for crystalline solids, but it works equally well for complex biomolecules using the supercell. The use of atomic orbitals in the basis expansion is particularly appropriate for such calculations. Details of the method can be found in Ref. 7. Vienna *ab-initio* simulation package (VASP)^37^,^38^ is also a DFT-based method used solely to relax the structure of the biomolecule. It uses plane waves as the basis set and is very efficient for geometry optimization and structural relaxation. In the present work, we used the PAW-PBE potential for the exchange-correlation functional^39^. The combination of these two methods enables us to investigate materials of very complex structures^27^.

The same OLCAO method is also used to calculate the imaginary part of the frequency-dependent dielectric response function 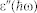 within the *random phase approximation* (RPA) of the one-electron theory according to the following equation (for details see Ref. 7): 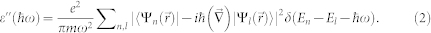

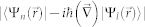
 is the dipolar matrix element of electron transition from the occupied molecular orbital state *Ψ_l_* to the state *Ψ_n_* with 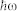 being the transition energy. 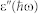 or its Kramers-Kronig transform enters the Lifshitz theory of vdW interactions^10^.

## Author Contributions

P.A. and W.Y.C. performed *ab initio* quantum mechanical calculations. N.F.S. suggested the system for study. P.A., A.W.M., R.H.F., V.A.P., N.F.S., R.P. and W.Y.C. contributed to the analysis and discussion of the paper, and edited the manuscript.
